# Stochastic Photoresponse‐Driven Perovskite TRNGs for Secure Encryption Systems

**DOI:** 10.1002/advs.202412139

**Published:** 2025-02-13

**Authors:** Dante Ahn, Minz Lee, Woochul Kim, Yeon Kyung Lee, Jun Young Lee, Gun Young Jung, Hangyeol Choi, Yohan Yoon, Hyun Seok Song, Heon Lee, Minah Seo, Jungwook Min, Yusin Pak

**Affiliations:** ^1^ Sensor System Research Center Korea Institute of Science and Technology (KIST) 14‐5 Hwarang‐ro Seoul 02792 Republic of Korea; ^2^ KU‐KIST Graduate School of Converging Science and Technology Korea University 145 Anam‐ro Seoul 02841 Republic of Korea; ^3^ Department of Materials Science and Engineering Korea University 145 Anam‐ro Seoul 02841 Republic of Korea; ^4^ Department of Biomicrosystem Technology Korea University 145 Anam‐ro Seoul 02841 Republic of Korea; ^5^ Diffusion Technology Team Memory Manufacturing Technology Samsung Electronics Co. Ltd. 145 Anam‐ro Seoul 02841 Republic of Korea; ^6^ School of Materials Science and Engineering Gwangju Institute of Science and Technology (GIST) 123 Cheomdangwagi‐ro Gwangju 61005 Republic of Korea; ^7^ Korea Aerospace University Department of Materials Engineering 76 Hanggongdaehak‐ro Goyang 10540 Republic of Korea; ^8^ Department of Optical Engineering Kumoh National Institute of Technology 350‐27 Gumi‐daero Gumi Gyeongsangbuk‐do Republic of Korea

**Keywords:** encryption, hybrid perovskite, NIST randomness test, stochastic photoresponse, true random number generator

## Abstract

True random numbers are essential for ensuring information security and supporting simulations across various industries. With the exponential growth of data driven by advancements in artificial intelligence, robust encryption for communications has become increasingly important. While software‐based deterministic random number algorithms are cost‐effective and easy to use, they are vulnerable to attacks by powerful supercomputers, highlighting the need for more secure alternatives. As portable electronic devices and information‐gathering sensors proliferate, portable true random number generators (TRNGs) are critical for maintaining security. In this work, hybrid material‐based photodetectors composed of anionic polymers and perovskites that maximize stochastic photogeneration for TRNG applications are presented. By integrating perovskite photodetectors with simple electronic circuits, compact, low‐power TRNG devices have been developed that are versatile and resilient to environmental factors. These devices generate 10 000 bits s^−1^ without resets or delays, achieving significant miniaturization. The generated 10 Mbit random number is validated through US National Institute of Standards and Technology (NIST) testing. Using a 480 000‐bit random sequence, perfect image encryption, ensuring protection against hacking are demonstrated. Additionally, the perovskite TRNG can operate under external light even when embedded in pork skin, realizing its potential as an implantable device for personal security and authentication.

## Graphical abstract

This study presents a hybrid anionic polymer and perovskite TRNG generating 10 000 bits s^−1^, validated by NIST testing. Exceptional image encryption and implantable potential are demonstrated, highlighting advancements in portable and implantable security devices.

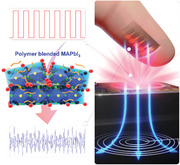

## 1 Introduction

Random numbers are integral to diverse fields such as lotteries, simulations, and research in mathematics and physics.^[^ ^1^, ^2^, ^3^ ^]^ However, their primary application lies in cryptographic protocols, particularly as the Internet of Things (IoT) and edge computing have dramatically increased the accessibility of personal information, heightening the need for robust encryption technologies.^[^ ^4^, ^5^, ^6^, ^7^, ^8^ ^]^

Despite advancements in digital security using biometric data like fingerprints and iris patterns, the vulnerabilities of software‐based pseudorandom number generators have driven interest in non‐deterministic TRNGs.^[^ ^9^, ^10^, ^11^, ^12^ ^]^ Unlike deterministic methods, photon‐based TRNGs rely on random physical phenomena based on entropy sources such as noise and particles, making their patterns nearly impossible to detect.^[^ ^13^, ^14^, ^15^ ^]^ However, these TRNGs are typically sensitive to environmental changes (temperature, voltage) and require complex comparator circuits to continuously adjust their thresholds.^[^ ^16^, ^17^, ^18^ ^]^

Accordingly, single‐photon‐based TRNGs, which simplify random number generation systems and maximize uncertainty, have attracted significant attention. By adopting timing principles, these TRNGs achieved impressive generation speeds of up to 145 million random numbers per second, reducing dependence on hardware performance (such as beam splitters and photodetectors) while enhancing speed. However, developing a compact and portable system remains a significant challenge.^[^ ^19^, ^20^, ^21^ ^]^

Recent studies have explored TRNGs based on diffusion‐type memristors to address miniaturization and stability by leveraging random variations in switching voltage, time, and conductance. While promising, this approach is limited by issues such as current drift, long switching delays, and frequent calibration, which hinder its suitability for high‐speed applications.^[^ ^22^, ^23^ ^]^ Advances using NbOx‐based Mott memristors and 2D insulating layers like h‐BN and Bi2O2Se have further improved miniaturization and stability, achieving speeds of up to 100 000 bits s^−1^. However, challenges such as thermal instability and the durability of the insulating layers persist.^[^ ^24^, ^25^ ^]^

**Figure 1:**
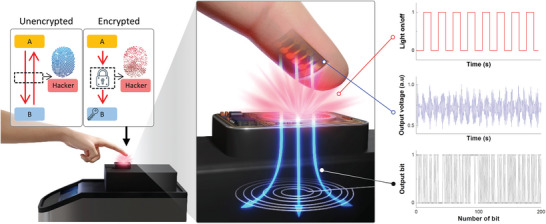
Illustration of a TRNG system for biometric implantable encryption and authentication, with a simple process for stochastic photovoltage generation and random number extraction.

In this study, we present a novel hybrid photodetector‐based TRNG that integrates anionic polymers and perovskites, offering a compact and efficient solution for TRNG. This device achieves a generation rate of 10 000 bits s^−1^ with high reliability, free from issues such as resets or time delays. Rigorous testing, including NIST randomness evaluations, confirms its performance across various operating conditions. The device demonstrates exceptional robustness, operating through pig skin and enduring environmental stressors such as vibrations, heat, and humidity. Furthermore, a randomly generated 480 000‐bit sequence successfully encrypted an original image, effectively preventing hacking attempts. These results highlight the potential for developing highly portable and implantable TRNGs, as illustrated in **Figure** **1**. Such advancements are poised to enhance computing and IoT security, setting a new standard for stability and reliability in modern technologies.

Hybrid organic‐inorganic perovskites exhibit excellent absorption coefficients across the wavelength range from ultraviolet to near‐infrared^[^ ^26^ ^]^ and are utilized as selective photodetectors due to their tunable absorption wavelengths achievable through simple solution processes. However, electrical and optoelectronic hysteresis, arising from factors such as ion migration, interfacial effects, and charge traps, results in relatively irregular outputs compared to all‐inorganic perovskites. These hysteresis effects are undesirable in phototransistors and solar cells but can be leveraged to enhance irregularities and randomness in TRNG operation.

## 2 Results and Discussion

**Figure 2:**
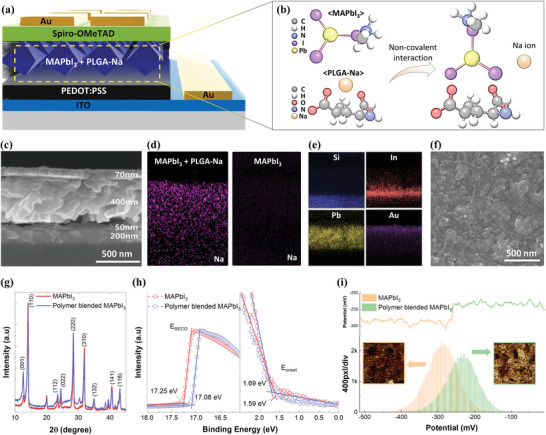
a) Schematic of the polymer blended perovskite device structure. b) Speculation on the molecular structural changes that occur when MAPbI_3_ and PLGA‐Na interact. c) Cross‐sectional SEM image of the polymer‐blended perovskite device. EDS results show the atomic distribution of d) Na, e) Si, In, Pb, and Au in the perovskite devices. f) Top‐view SEM image of the perovskite film. g) GIXRD results of the devices for crystallinity analysis. h) UPS results verifying the *E*_F_ and *E*_VBM_ levels. i) KPFM results of MAPbI_3_ films with and without the blended polymer, showing the contact potential difference.

**Figure** **2**a shows the structure of the polymer‐blended perovskite device, consisting of a PEDOT:PSS layer on an ITO/PET substrate, a poly‐L‐glutamic acid monosodium salt (PLGA)‐PbI_2_ thin film converted to MAPbI_3_, a Spiro‐OMeTAD hole transport layer, and a 70 nm gold electrode (Figure S1, Supporting Information). It is hypothesized that the non‐covalent interactions between MAPbI_3_ and PLGA‐Na do not significantly disrupt the perovskite crystal structure, as indicated in Figure 2b. This suggests that while these materials may interact, the overall integrity of the crystal lattice remains largely intact. Figure 2c–f shows scanning electron microscope (SEM) images and the energy‐dispersive spectroscopy (EDS) results, verifying the uniform stacking of layers and atomic distribution in the PLGA‐MAPbI_3_ film (hereafter, referred to as the polymer‐blended perovskite). In our fabrication, the film quality of the polymer‐blended perovskite was better than that of the bare film (Figure S2, Supporting Information). Literature suggests that Lewis bases, such as PLGA, are effective in slowing down perovskite crystallization, thereby facilitating the formation and uniform distribution of crystals.^[^ ^27^ ^]^

The impact of polymer blending on the crystallinity of the perovskite was further confirmed by grazing incidence X‐ray diffraction (GIXRD) analysis, as shown in Figure 2g. Variations in peak intensity serve as indicators of alterations in atomic configuration and crystallinity. For the polymer‐blended perovskite, a notable increase in the intensity of the (001) and (110) peaks from 0.146 to 0.253 was observed, suggesting that the incorporation of PLGA facilitates the preferential growth of specific crystal facets of MAPbI_3_, potentially leading to more uniform and orderly grains.^[^ ^28^, ^29^ ^]^ Conversely, the intensities of other peaks, specifically (112), (022), (310), (141), and (116), were slightly reduced. In this work, we employed the so‐called two step conversion process (PbI_2_→MAPbI_3_), and the results indicate that the blended polymer has minimal impact on the formation of PbI_2_ precursor (Figure S3, Supporting Information).

Ultraviolet photoelectron spectroscopy (UPS) was conducted to assess the energy band structures of polymer‐blended perovskite films. The Fermi level positions for the MAPbI_3_ films, with and without polymer blending, were measured to be −4.14 and −3.97 eV, respectively, utilizing the equation for Fermi energy (*E*_F_) = 21.22 – *E*_SECO_, where *E*_SECO_ represents the secondary electron cutoff energy (Figure 2h).^[^ ^30^, ^31^ ^]^ The valence band maxima (VBM) were calculated to be −5.66 eV for the bare film and −5.73 eV for the blended film, using the equation *E*_VBM_ = 21.22 – (*E*_SECO_ – *E*_onset_), where *E*_onset_ denotes the onset of electron energy. The shifts in band energy levels reconfirm changes in the optoelectronic and charge transport properties of the polymer‐blended perovskite.

We conducted Kelvin probe force microscopy (KPFM) on both the control and the polymer‐blended perovskite to measure the contact potential difference (CPD), as shown in Figure 2i. The grain size and morphology showed minimal changes, consistent with the SEM images discussed above. However, the surface contact potential of the polymer‐blended perovskite film exhibited an increased peak value (over 70 mV) compared to the control sample. We attribute this increased potential to changes in surface and sub‐surface charge concentration induced by the blended polymer, indicating the occurrence of charge carrier trapping and non‐radiative recombination.^[^ ^32^ ^]^

As shown in Figure 2, the defect states caused by the blended polymer can lead to variations and randomness in the intensity and periodicity of photogenerated output voltage. We compared the photovoltage characteristics of the polymer‐blended perovskite with those of a bare perovskite (with low defect concentration; see Figure S4, Supporting Information) and commercial single‐crystal silicon (Si) photodiode (with minimal defects; see Figure S5, Supporting Information). The presence of series resistance (*R_s_*) can hinder the separation and extraction of photo‐generated charges in a photodiode. *R_s_* is determined by analyzing deviations in the *I*–*V* characteristics from ideal diode behavior, particularly in the forward bias region under illumination, where these deviations indicate voltage losses caused by *R_s_* for a given current.

**Figure 3:**
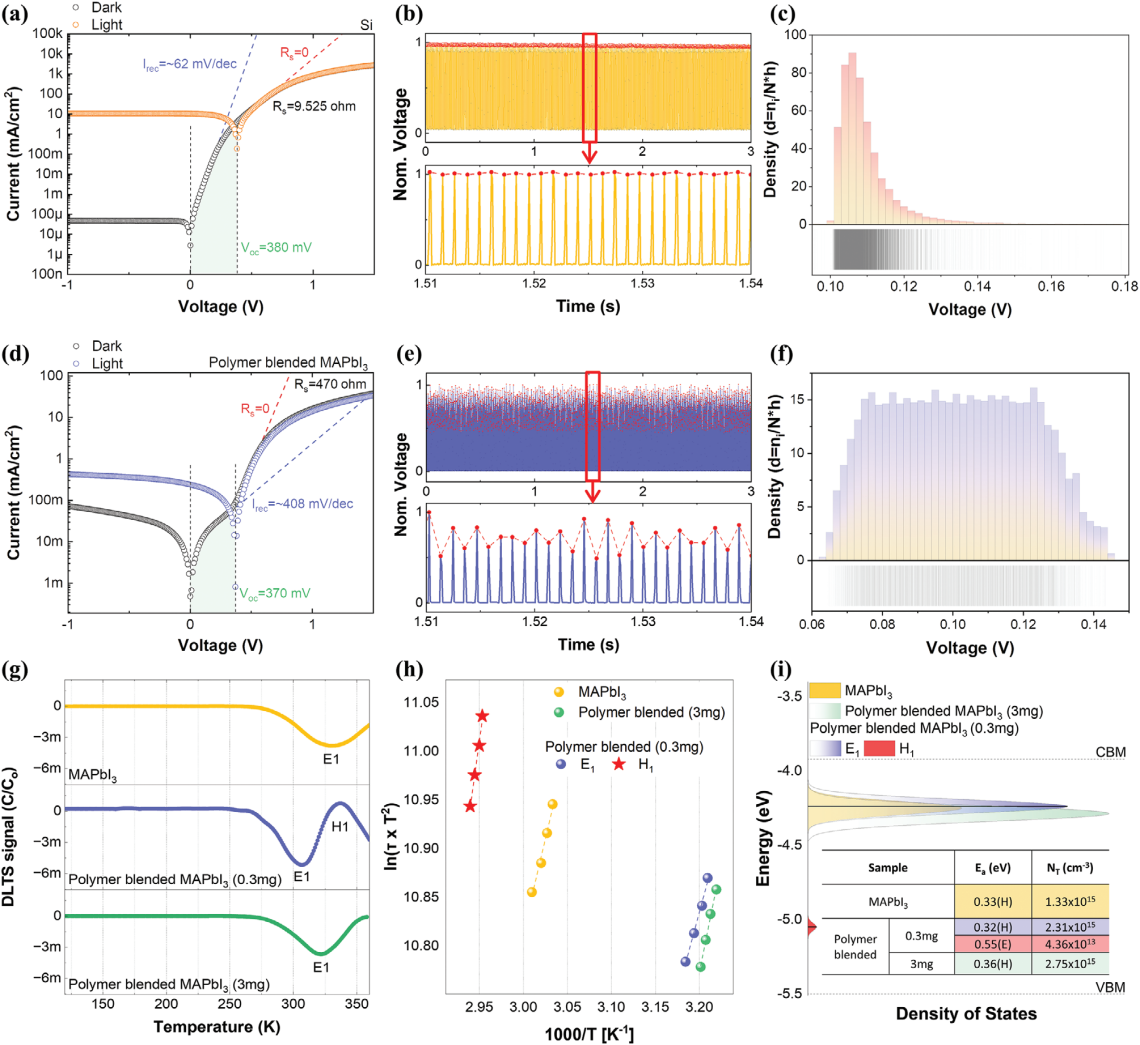
a) Current‐voltage (*I*–*V)* curves, b) voltage variation, and c) histogram of output photovoltages, measured in the Si photodiode. d) *I*–*V* curves, e) voltage variation, and f) histogram of output photovoltages, measured in the polymer‐blended perovskite device. g) DLTS signals and h) Arrhenius plots of DLTS signals from the samples of bare MAPbI_3_, 3 mg polymer‐blended MAPbI_3_, and 0.3 mg polymer‐blended MAPbI_3_, respectively. i) Density of trap states within the band gap, as extracted from the DLTS measurements.

**Figure** **3** shows the electronic and optoelectronic characteristics of the Si photodiode and polymer‐blended perovskite device under 450 nm, 7 mW light. The open‐circuit voltage (*V*_oc_) measured was 380 mV for the Si photodiode, 370 mV for the polymer‐blended MAPbI_3_, and 220 mV for the bare MAPbI_3_ (Figure S4, Supporting Information). The influence of *R*_s_ on photo‐generation can be assessed by comparing the device performance to an ideal case where *R*_s_ = 0, as illustrated by the red dotted lines in Figure 3a,d. Specifically, the *R*_s_ values were found to be 9.525 Ω for the Si photodiode and 470 Ω for the polymer‐blended perovskite device. Carrier recombination properties related to random variations in output photovoltage were analyzed through recombination currents (*I*_rec_), marked by an initial steep rise in dark current (blue dotted lines in Figure 3a,d). The Si photodiode had a recombination current of 62 mV·dec^−1^, while the polymer‐blended perovskite device showed 408 mV·dec^−1^, indicating the wider distribution of output photovoltages in the polymer‐blended perovskite device (Figure S6, Supporting Information).

Figure 3b,e shows the output photovoltage characteristics of the Si photodiode and the polymer‐blended perovskite device, respectively, measured using a 450 nm, 7 mW light source with a 2 ms on/off cycle. Normalized output voltages (represented by red dots) in the Si photodiode converge to nearly one, while the polymer‐blended perovskite device exhibits irregularly fluctuating output voltages. To visually represent the density of data points within specific voltage ranges, peak values were extracted and displayed as histograms in Figure 3c,f. Photovoltage fluctuations in the Si photodiodes typically occur within 0.15 V, with the most frequent values (highest densities) ranging between 0.94 and 0.96 V. In contrast, the polymer‐blended perovskites exhibit fluctuations between 0.4 and 1 V, with data points randomly distributed. This indicates that a fully probabilistic photogeneration process is occurring, beyond the predictable range where no discernible pattern exists. We speculate that the presence of PLGA, which contains negatively charged carboxylate groups, can form non‐covalent bonds with Pb^2+^ ions in MAPbI_3_. These interactions can distort the lattice structure and impede charge carrier mobility, leading to reduced electrical conductivity and resulting in irregular voltage output.

To investigate the origin of the increased photovoltage variability in the polymer‐blended perovskite device, deep level transient spectroscopy (DLTS) measurements were conducted.^[^ ^33^, ^34^ ^]^ For comparison, MAPbI_3_ was blended with varying amounts of PLGA polymers (20, 3, and 0.3 mg), with pristine MAPbI_3_ used as a reference. It is important to note that DLTS measurements could not be conducted on the 20 mg polymer‐blended MAPbI_3_ sample due to unstable capacitance values, which were insufficient to observe the capacitance decay required for recording DLTS signals.

Figure 3g presents the DLTS spectra of the three samples across a temperature range of 120–360 K. All samples exhibited an E1 peak, corresponding to a negatively charged defect in MAPbI_3_, detected between 307 and 331 K (**Table** **1**). The 0.3 mg polymer‐blended sample additionally displayed an H1 peak at 337 K, which is attributed to a positively charged defect. In contrast, the H1 peak was absent in the other samples. The absence of the H1 peak in the 3 mg polymer‐blended sample may be attributed to the increased polymer content, potentially leading to agglomeration that prevents the defects from being activated to move around. It is important to note that the defects observed in perovskite materials by DLTS are more closely related to mobile ions rather than electronic defects, as these materials contain a significant number of mobile halide ions when a bias voltage is applied.^[^ ^35^, ^36^ ^]^

**Table 1:** Defect parameters of the samples obtained by DLTS measurements.

Sample	Defect states and peak temperature [K]	Activation Energy, E_a_ [eV]	Doping density, N_D_ [cm^−3^]	Trap density, N_T_ [cm^−3^]	Defect origin
MAPbI_3_	E1 (331.10)	E_C_−0.32	1.75 × 10^16^	1.33 × 10^15^	I^–^
Polymer‐blended MAPbI_3_ (3 mg)	E1 (322.79)	E_C_−0.33	3.00 × 10^16^	2.79 × 10^15^	I^–^
Polymer‐blended MAPbI_3_ (0.3 mg)	E1 (307.79)	E_C_−0.36	2.56 × 10^16^	2.31 × 10^15^	I^–^
	H1 (337.18)	E_V_+0.55		4.36 × 10^13^	MA^+^ or polymer induced defect

Defect parameters, including activation energy (*E*_a_) and defect density (*N*_T_), were derived from Arrhenius plots of the DLTS signals. Figure 3h presents the Arrhenius plot corresponding to the DLTS signals, with the evaluated doping density (*N*_D_), *E*_a_, defect state positions, and *N*_T_ summarized in Table 1. The *N*_D_ and *E*_a_ values for the E1 peak were found to be ≈10^16^ cm^−3^ and *E_C_* – (0.32–0.36) eV, respectively, indicating similar doping concentrations across the MAPbI_3_ samples, with the presence of intrinsic defects. We attribute the intrinsic defect E1 to mobile iodine ions.^[^ ^37^ ^]^ While all samples exhibited the intrinsic E1 defect peak, variations in concentration were observed. The polymer‐blended MAPbI_3_ sample showed an E1 defect concentration twice that of the pristine MAPbI_3_ sample, suggesting that polymer incorporation may enhance E1 defect formation. Additionally, the H1 peak at *E*_V_ + 0.55 eV observed in the 0.3 mg polymer‐blended MAPbI_3_ sample may be attributed to mobile MA^+^ or polymer induced defects.^[^ ^38^ ^]^ The positions and densities of traps or defects induced by the polymer are illustrated in Figure 3i. The 0.3 mg polymer‐blended MAPbI_3_ sample contains both positively charged defects (H1) and negatively charged defects (E1).^[^ ^39^ ^]^ We speculate that the interaction between oppositely charged defects (E1 and H1) under light‐induced electric fields leads to trap‐assisted charge trapping and release, resulting in a non‐linear and stochastic photoresponse.^[^ ^40^ ^]^ This behavior induces photovoltage fluctuations at the microscale. The increased concentration of E1 and H1 defects in the polymer‐blended MAPbI_3_ sample enhances the dynamic modulation of the local electric field, improving randomness‐driven performance. These results underscore the polymer's critical role in tuning defect densities and their influence on charge dynamics.

**Figure 4:**
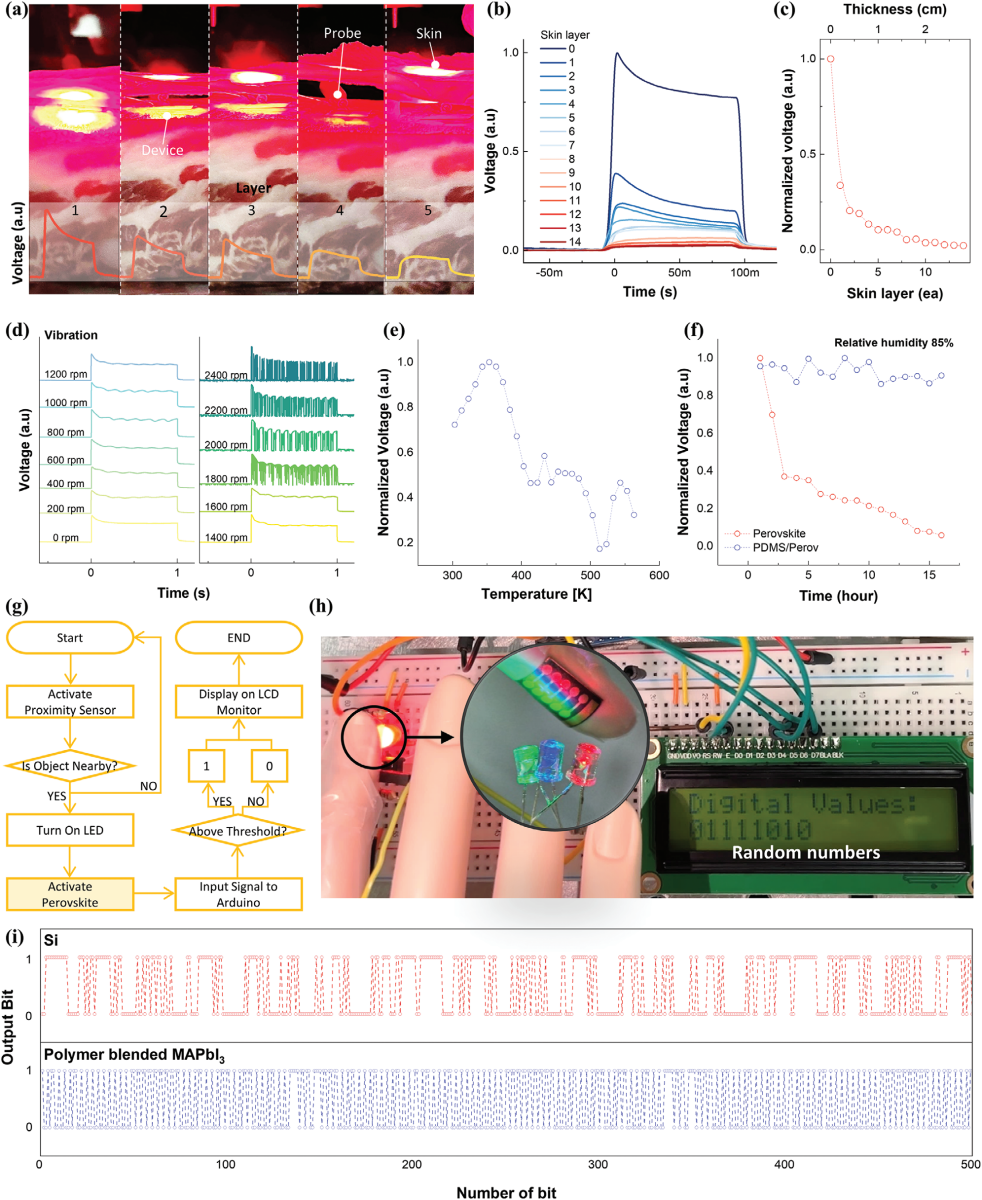
a) Bio‐implantable test: light transmission and photovoltaic generation with varying pig skin thickness. b) Photovoltages generated across increasing skin layers. c) Normalized photovoltages versus skin thickness and the number of layers. Verification of photovoltage generation under different real‐world conditions, such as d) vibration, e) temperature, and f) humidity. g) TRNG operation protocol and h) a real streamlined TRNG circuit with the smallest possible components so far, demonstrating real‐time TRNG. i) Output bits corresponding to random numbers extracted from TRNGs using the control Si photodiode and the polymer‐blended perovskite device (see Figure S7, Supporting Information for the output bits data of the bare perovskite device).

The principle of irregular generation and stochastic distribution of output photovoltages resulting from the polymer blending approach is clearly demonstrated above. Before developing a bio‐implantable TRNG, we assessed whether factors such as vibration, temperature, humidity, and light penetration through the skin could interfere with TRNG operating characteristics. To ensure light could penetrate the epidermis and properly reach the TRNG, we tested the device by embedding it in pork skin and flesh, which closely resemble human skin, at depths of up to 2 cm, as shown in **Figure** **4**a. Figure 4b,c presents the results from the pork skin test. The light was irradiated while incrementally increasing the number of skin layers (0.2 cm per layer). No measurable photovoltage was produced beyond 15 layers of pork skin, corresponding to a maximum thickness of 3 cm. However, up to five layers, corresponding to a thickness of 1 cm, sufficient photovoltage was generated to operate the TRNG.

Further tests were performed to evaluate the effects of vibration, temperature, and humidity on the irregular photovoltage generation, as illustrated in Figure 4d–f. The photovoltage remained consistent up to a rotation speed of 1600 rpm on the vibration generator. Although the output became unstable at higher vibration levels, specifically above 1800 rpm, the amplitude of the photovoltage was preserved. This suggests that the output bits generated under vibration conditions at 2400 rpm could exhibit enhanced stochasticity and irregularity. Temperature tests were conducted across a range of 300–570 K, during which photovoltage generation was consistently maintained, indicating robust performance within the physiological temperature range. Under 85% relative humidity, perovskite degradation was observed; however, stability was effectively enhanced by applying a passivation layer of PDMS a few microns thick. We assessed factors like skin interference, heat, vibration, temperature, and humidity in implantable scenarios. For long‐term use, it is crucial to examine the impact of physical activities, immune responses, mechanical shocks, and bodily fluids on device stability and durability. Further research is needed to address these influences.

The operation of the TRNG, designed to generate non‐hackable bits, follows the protocol outlined in Figure 4g. When an object approaches the proximity sensor, the LED blinks, and the flexible perovskite device generates irregular photovoltages. These voltages are processed by an Arduino, which then displays either 1 or 0 on the LCD based on a predefined threshold value. Apart from the perovskite device in Figure 4g, the other components can be miniaturized and powered with low energy consumption using the latest technology. Figure 4h shows a photograph of the TRNG circuit to demonstrate the generation of continuous output bits. We verified the scenario where the polymer‐blended perovskite device attached to a mannequin's finger received external LED light to induce photovoltages and generate unhackable bits in the TRNG circuit. The TRNG system comprises four components: a light‐emitting diode (LED), a photodiode, an amplifier, and a JK flip‐flop, representing a notably simplified circuit configuration (Figure S8, Supporting Information). A small electrical signal detected by the polymer‐blended perovskite device is amplified and then used as a clock signal for the JK flip‐flop. The output voltage from the JK flip‐flop is designated as a bit, with values above 0.5 V assigned as ‘1′ and those below 0.5 V as ‘0′. Each on/off light signal cycle generates one bit.

Figure 4i shows a fraction of the binary outcomes (200 out of 10^6^, Figure S7, Supporting Information). To accumulate 10^6^ bits, the LED was activated at a frequency of 10 kHz over a duration of 500 s. The Si photodiode‐based TRNG generated up to 100 random numbers per second with 10 ms pulses, while the polymer‐blended perovskite TRNG produced 10 000 random numbers per second with a faster 100 µs pulse input. (Figures S9–S11, Supporting Information). To verify the randomness of the generated binary sequences, we conducted the NIST randomness evaluation (*NIST 800–22*) as shown in **Table** **2**, which encompasses 15 distinct statistical tests (Figure S12, Supporting Information for NIST test details). We collected a total of 1 million bits for each NIST test (Figures S12–S15, Supporting Information). If the p‐value exceeds 0.01, the test is considered passed, suggesting that the dataset is more randomly distributed as the p‐value increases (approaching 1).^[^ ^41^ ^]^ Compared to the Si and bare perovskite TRNGs, the polymer‐blended perovskite TRNG surpassed all 15 tests with an average pass rate above 97%, solidifying the generated number sequence as nearly unpredictable and devoid of discernible correlation.

**Table 2:** NIST randomness test results in this study.

	Test	Si		MAPbI_3_		Polymer blended MAPbI_3_	
		P‐VALUE	Result	P‐VALUE	Result	P‐VALUE	Result
1	Frequency (monobit) test	–	Failed	0.213309	Passed	0.739918	Passed
2	Frequency test within a block	–	Failed	0.122325	Passed	0.534146	Passed
3	Cumulative sums (cusum) test	–	Failed	0.534146	Passed	0.911413	Passed
4	Runs test	–	Failed	0.035174	Passed	0.798336	Passed
5	Test for the longest run of ones in a block	–	Failed	–	Failed	0.579443	Passed
6	Binary matrix rank test	0.039105	Passed	0.350485	Passed	0.693720	Passed
7	Discrete Fourier transform (spectral) test	0.000954	Failed	0.122325	Passed	0.534146	Passed
8	Non‐overlapping template matching test	0.035174	Passed	0.066882	Passed	0.739918	Passed
9	Overlapping template matching test	0.017912	Passed	0.000439	Failed	0.627132	Passed
10	Maurer's “universal statistical” test	–	Failed	–	Failed	0.350485	Passed
11	Approximate entropy test	–	Failed	0.035174	Passed	0.534146	Passed
12	Random excursions test	–	Failed	–	Failed	0.397227	Passed
13	Random excursions variant test	–	Failed	–	Failed	0.855265	Passed
14	Serial test	–	Failed	0.004301	Failed	0.711770	Passed
15	Linear complexity test	0.000439	Failed	0.350485	Passed	0.739918	Passed

**Table** **3** compares hardware random number generators, including semiconductor and quantum‐based systems. The polymer‐blended MAPbI_3_ TRNG developed in this study shows advantages in miniaturization, speed (10 K bit rate), and portability, with a NIST pass rate of over 97%. While it surpasses many semiconductor systems, QRNGs offer superior randomness and speeds but require complex setups like cryogenics. Further optimization is needed to improve the MAPbI_3_ TRNG's randomness and speed to approach quantum system capabilities.

**Table 3:** NIST test results in the relevant literature.

	Hardware resource	Material	Bit operation rate[bs^−1^]	NIST test	Required Circuit	Portable	Pass rate	Refs.
1	Quantum	polymer‐blended MAPbI_3_	10 K	15/15	4	O	> 97%	This work
2	Quantum	MAPbI_3_	10 K	9/15	4	O	> 59%	This work
3	Quantum	Si	1 K	3/15	4	O	> 32%	This work
4	Electrical	*h*‐BN	5	15/15	6	O	–	[25]
5	Thermal	NbO_x_	100 K	15/15	4	O	> 99%	[24]
6	Electrical	HfO_2_	6 K	8/15	4	O	> 99%	[42]
7	Quantum	Single photon detector	100 K ∼	16/16	10	X	> 98%	[43]
8	Quantum	InGaAs/InP	23	15/15	14	X	> 99%	[44]
9	Quantum	Transition‐edge‐sensor	0.4	15/15	19	X	–	[45]
10	Quantum	Superconducting nanowire	114	15/15	33	X	> 99%	[20]
11	Quantum	Single photon detector	1 M∼	15/15	7∼	X	> 94%	[46]
12	Quantum	Single photon detector	≈18.8 G	15/15	18	X	–	[19]

The discussion above thoroughly validated the irregular photo‐voltage generation properties suitable for in vivo implant environments, the fabrication of a truly miniaturizable random number generation system based on these properties, and the randomness of the generated numbers. We further studied a cryptographic application to assess whether the generated random numbers could be effectively utilized in security protocols.

**Figure 5:**
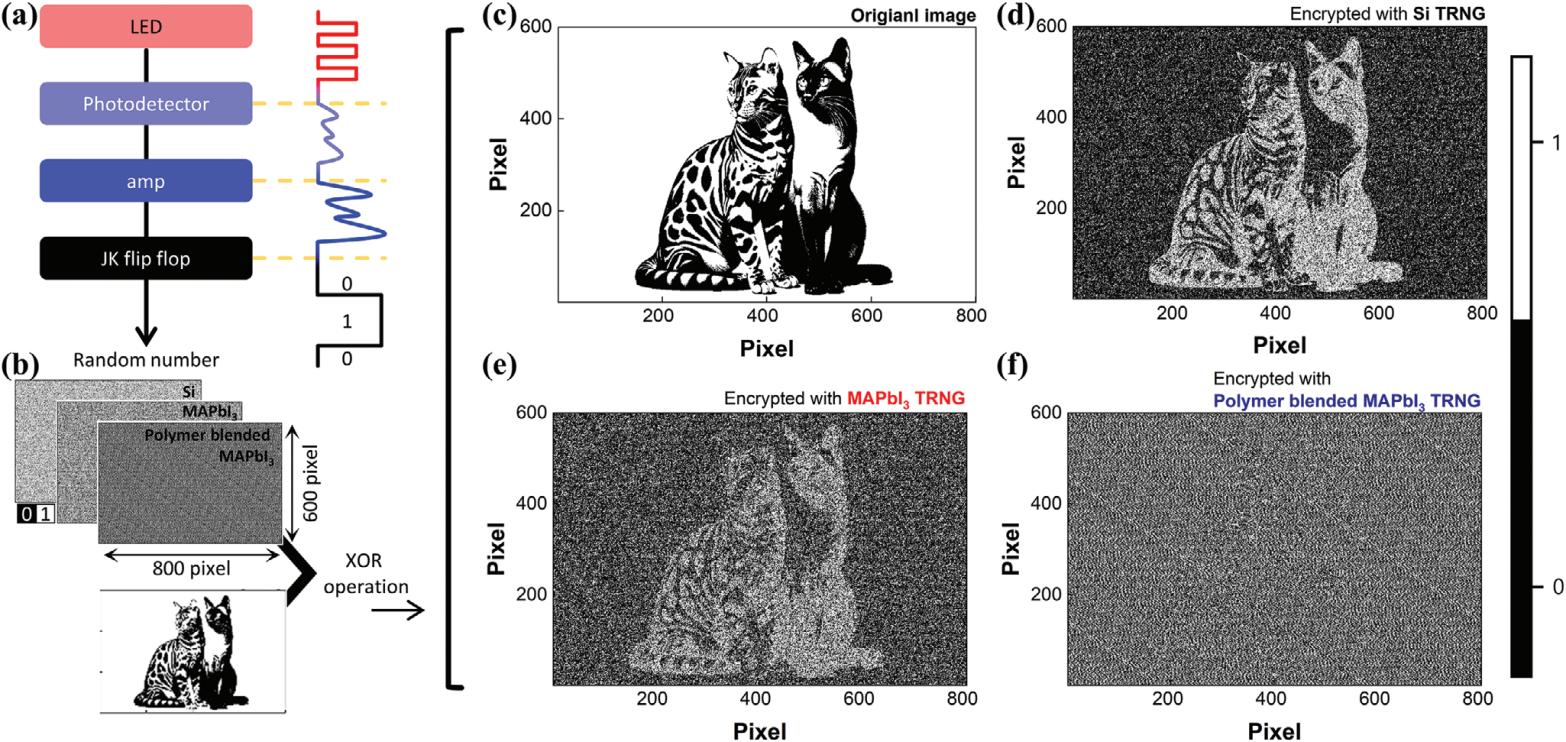
a) Random number extraction process signal flowchart. b) 480 000‐bit random numbers extracted using each device (Si, MAPbI_3_, and polymer‐blended MAPbI_3_) and XOR operation with c) the original image. d–e) Images XOR‐operated with hackable random numbers generated by the Si TRNG and MAPbI_3_ TRNG, respectively. f) Image XOR‐operated with unhackable true random numbers generated by the polymer‐blended MAPbI_3_ TRNG.

**Figure** **5** shows the result of encrypting an original image through an XOR operation using random numbers generated under the previously demonstrated skin scenario. Commercial Si, bare perovskite, and polymer‐blended perovskite devices were connected and tested respectively. The random number extraction process is illustrated in Figure 5a. First, 7 mW light emitted from an LED is incident on the photodetector, generating an output voltage. This generated voltage is amplified and sent to the JK flip‐flop, extracting digital bits of 0 and 1. A total of 480 000 bits of random numbers were extracted in this way to proceed with the encryption of an image consisting of 600 × 800 pixels as shown in Figure 5b. The entire image converted from the 480 000 bits can be found in Figure S16 (Supporting Information). The XOR operation is a logical operation that outputs 0 when inputs A and B are the same and 1 when they are different, allowing us to confirm the encryption function when random numbers are calculated with the original image that has specific patterns. The original image used to confirm the encryption function is shown in Figure 5c. Figure 5d–f are images encrypted by the XOR operation with random numbers extracted using silicon, MAPbI_3_, and polymer‐blended MAPbI_3_ photodetectors, respectively.

## 3 Conclusion

The polymer‐blended MAPbI_3_ TRNG demonstrates exceptional encryption performance, achieving highly secure and non‐hackable encryption by leveraging the stochastic photoresponse of photoelectric materials. Compared to complex and large‐scale optical or quantum systems, our solution‐processed device offers compactness, scalability, and potential for cost‐effective mass production, making it a practical alternative for real‐world applications (refer to the Figure S17, Supporting Information). With continued advancements, it has the potential to become a key technology for next‐generation cryptographic applications, such as image encryption and secure communication protocols.

In conclusion, we have developed a novel hybrid photodetector‐based TRNG using anionic polymers and perovskites, achieving a significantly miniaturized perovskite TRNG that generates 10 000 bits s^−1^ without resets or delays. The TRNG demonstrated strong performance in data security, successfully encrypting an image using 480 000 bits of true random numbers. Additionally, its potential for implantable applications was validated through experiments with pork skin layers up to 1 cm thick, simulating the conditions of human skin implantation. Compared to commercial Si photodiodes, our TRNG increased the bit generation rate by more than 100 times without requiring standby voltage. NIST evaluations confirmed the high quality and reliability of the random numbers produced by the polymer‐blended perovskite TRNG. These results pave the way for the development of portable and implantable electronic devices and sensors, offering enhanced security and encryption for personalized applications.

## 4 Experimental Section

4.1

4.1.1

##### Fabrication of the MAPbI_3_ Device

The MAPbI_3_ device was fabricated on a 200 nm ITO‐coated PET substrate (1–10 Ω cm^−1^), cleaned with acetone and isopropyl alcohol, followed by UV ozone treatment for 30 min. A PEDOT:PSS layer was spin‐coated at 5000 rpm for 40 s, and PbI_2_ (1.9 m in DMSO:DMF = 0.8:0.2) was deposited at 2000 rpm for 20 s. For the polymer blend sample, 0.3 mg of PLGA polymer was added to the PbI_2_ precursor. The MAPbI_3_ film was formed by spin‐coating an MAI solution (40 mg mL^−1^ in IPA with 0.9 vol% DMF) onto PbI_2_ at 3000 rpm for 30 s. A spiro‐OMeTAD hole transport layer (0.06 m in chlorobenzene with additives) was spin‐coated at 2000 rpm for 20 s. Gold electrodes were thermally evaporated at 1 Å/s. All steps were performed in a nitrogen‐filled glove box.

##### Thin Film Characterization

Cross‐sectional scanning electron microscope (SEM) imaging and energy dispersive X‐ray spectroscopy (EDS) analysis were carried out to comprehensively characterize the thin films. Grazing incidence X‐ray diffraction (GIXRD) measurements were performed at a 1.5° incidence angle using a Bruker D8 Advance instrument, providing insights into the crystallographic structure of the materials. Ultraviolet photoelectron spectroscopy (UPS) analysis was performed using a Nexsa (ThermoFisher Scientific) equipped with a He‐I (21.22 eV) excitation source, maintaining a base pressure of 2.0 × 10^−8^ mBar and a beam spot size of 1.0 mm × 1.0 mm. The work functions of the materials were obtained with a pass energy of 1.0 eV and an applied bias of −10 V.

##### Electrical Characterization

The optical response characteristics of the device were meticulously examined using a ROHDE & SCHWARZ RTM3004 oscilloscope by illuminating it with 450 nm monochromatic light from a Mightex SLC‐AV04‐US through a 100 µm diameter optical fiber. Additionally, DC *I*–*V* characteristics were measured using a Keithley 2636B source meter. Texas Instruments components SN74LS73AN for the JK flip‐flop and NE55322P for the amplifier were chosen to ensure accuracy and reliability in the extraction of random numbers.

##### DLTS Measurement

DLTS measurements were performed using a cryogenic probe station (Lakeshore TTPX). The capacitance was measured using an impedance analyzer (Zurich Instruments MFIA). DLTS measurements were performed over a temperature range of 120 K to 360 K, with a step size of 1 K, averaging 10 data points at each temperature. Capacitance‐voltage (*C‐V*) measurements were conducted at a frequency of 200 kHz. The reverse bias was set to −0.5 V and the forward bias was set to 0 V. The filling pulse width and pulse period width were 1 s, and 4 s, respectively.

## Conflict of Interest

The authors declare no conflict of interest.

## Supporting information



Supporting Information

## Data Availability

The data that support the findings of this study are available from the corresponding author upon reasonable request.
