# Supplementary material for: Stochastic Photoresponse‐Driven Perovskite TRNGs for Secure Encryption Systems
Source: Adv Sci (Weinh). 2025 Feb 13;12(15):2412139. doi: 10.1002/advs.202412139 (PMC12005754; doi:10.1002/advs.202412139)
Supplement: Supplementary file 1 — Supporting Information [file ADVS-12-2412139-s001.docx]

**Supporting Information**

**Stochastic Photoresponse-Driven Perovskite TRNGs for Secure Encryption Systems**

*Dante Ahn^1,3,+^, Minz Lee^1,4,+^, Woochul Kim^1^,* *Yeon Kyung Lee^1,5^, Jun Young Lee^6^, Gun Young Jung^7^,* *Hangyeol Choi^8^, Yohan Yoon^8^, Hyun Seok Song^1,3^, Heon Lee^4^, Minah Seo^1, 3^ Jungwook Min^2,*^ and Yusin Pak^1,*^*

+ Equally contributed to this work.

**^1^**Sensor System Research Center, Korea Institute of Science and Technology (KIST), Seoul 02792, Republic of Korea

*****E-mail: yusinpak@kist.re.kr

**^2^**Department of Optical Engineering, Kumoh National Institute of Technology, Gumi, 39253, Republic of Korea

*****E-mail: jungwook.min@kumoh.ac.kr

**^3^**KU-KIST Graduate School of Converging Science and Technology, **^4^**Department of Materials Science and Engineering, **^5^**Department of Biomicrosystem Technology, Korea University, Seoul, 02841, Republic of Korea

**^6^**Diffusion Technology Team, Memory Manufacturing Technology, Samsung Electronics Co. Ltd.

^7^School of Materials Science and Engineering, Gwangju Institute of Science and Technology (GIST), Gwangju 61005, Republic of Korea

**^8^**Korea Aerospace University, Department of Materials Engineering, Goyang, Republic of Korea

**Keywords:** true random number generator, hybrid perovskite, stochastic photoresponse, encryption, NIST randomness test

**Section Contents**

Section S1. Fabrication of the MAPbI_3_ device

Section S2. SEM images of the bare perovskite device

Section S3. GIXRD analysis of PbI_2_ and MAPbI_3_ films

Section S4. *I-V* curve of bare MAPbI_3_

Section S5. Specifications of the Commercial SLD-70BG2 Si Photodiode

Section S6. Output voltage distribution

Section S7. Output bits generated by the TRNGs

Section S8. Circuitry of our TRNG system

Section S9. Frequency dependence test for irradiation conditions

Section S10. Rising and falling time of the devices

Section S11. Clocking speed of the devices

Section S12. NIST randomness test

Section S13. 10^6^ bit random numbers generated from the Si TRNG

Section S14. 10^6^ bit random numbers generated from the bare MAPbI_3_ TRNG

Section S15. 10^6^ bit random numbers generated by the polymer-blended MAPbI_3_ TRNG

Section S16. 480 000 random numbers extracted from TRNGs

Section S17. Comparison of TRNG and QRNG Devices: Scalability, Integration, and Economic Feasibility

**Section S1. Fabrication of the MAPbI_3_ device**

**
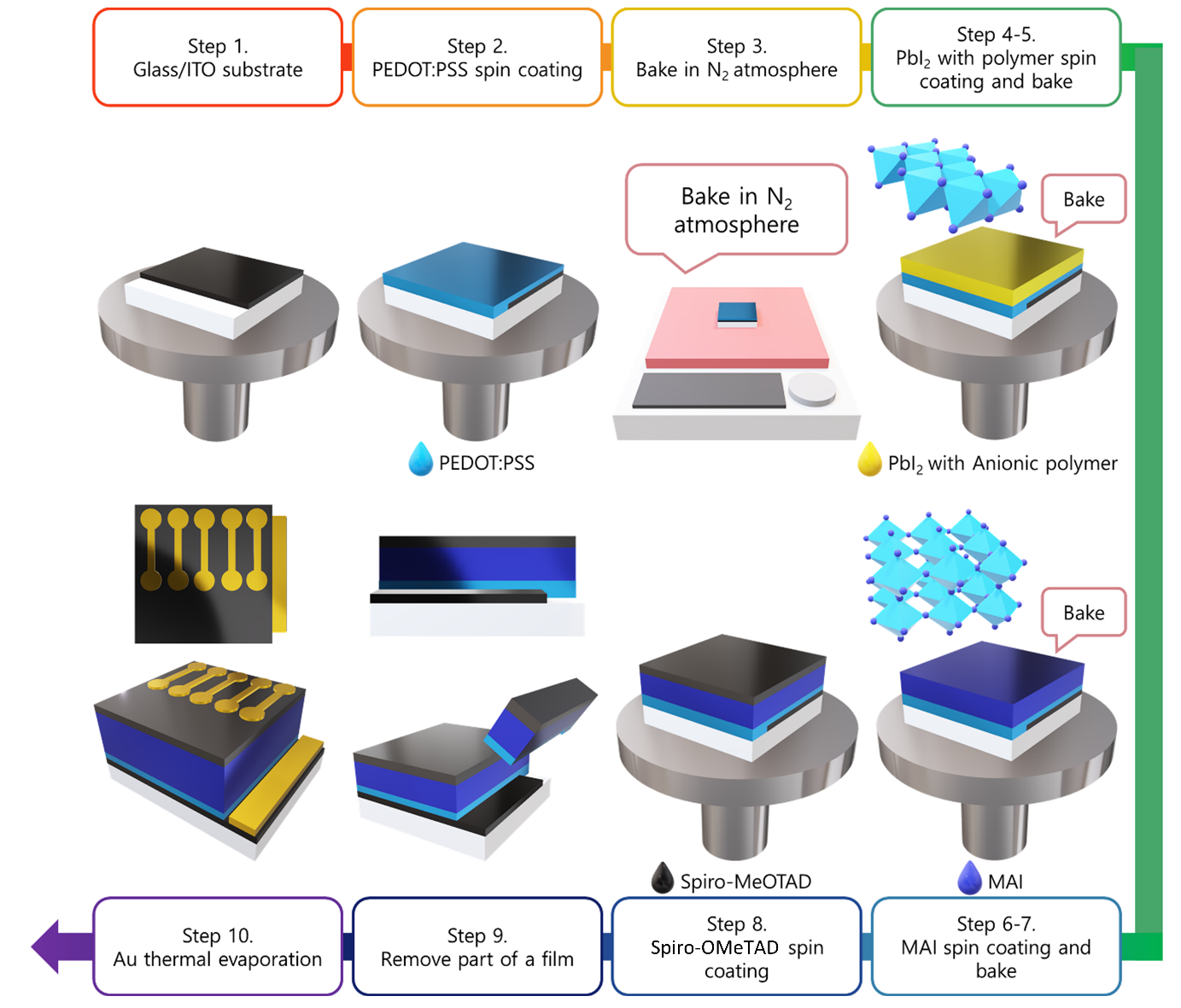
**

**Figure S1.** Schematic of the fabrication sequence for the MAPbI_3_ device to be applied in the TRNG system. The anionic polymer PLGA was added to the PbI_2_ precursor solution and stirred at 50 °C for 2 hours to facilitate blending.

**Section S2. SEM images of the bare perovskite device**

**
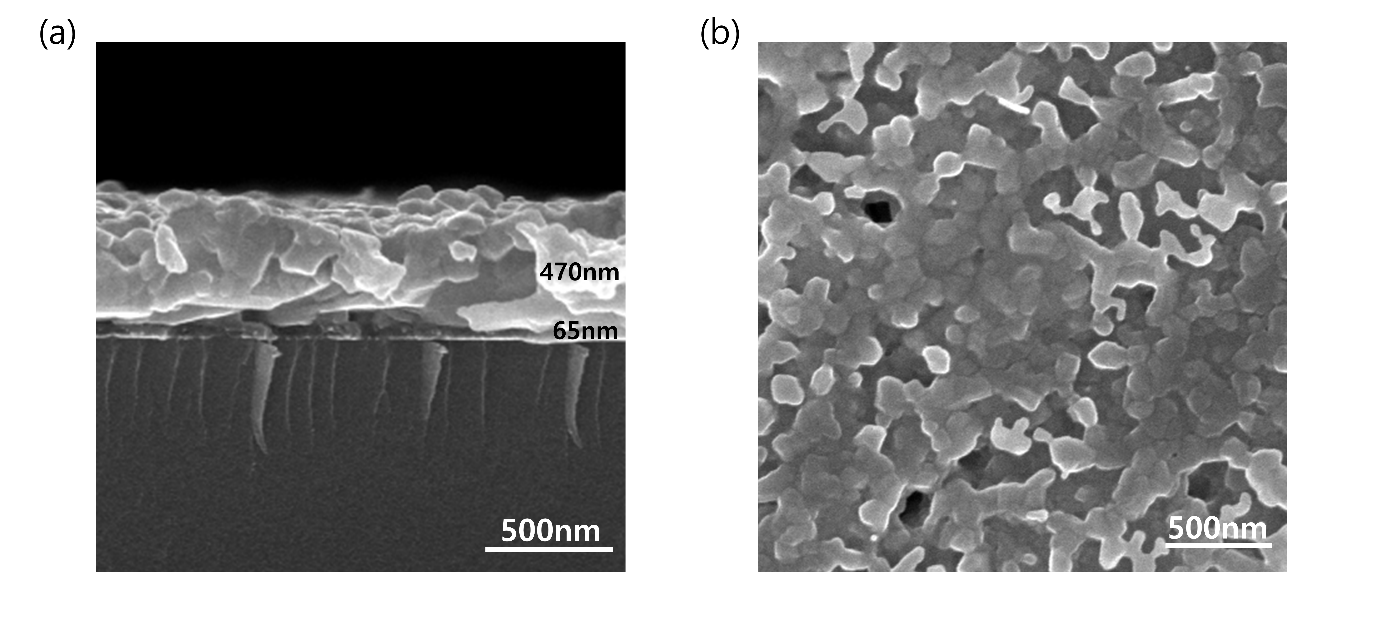
**

**Figure S2.** Scanning electron microscopy images of the bare perovskite device captured in (a) cross-sectional view and (b) top-view.

**Section S3.** **GIXRD analysis on PbI_2_ and MAPbI_3_ films**

**
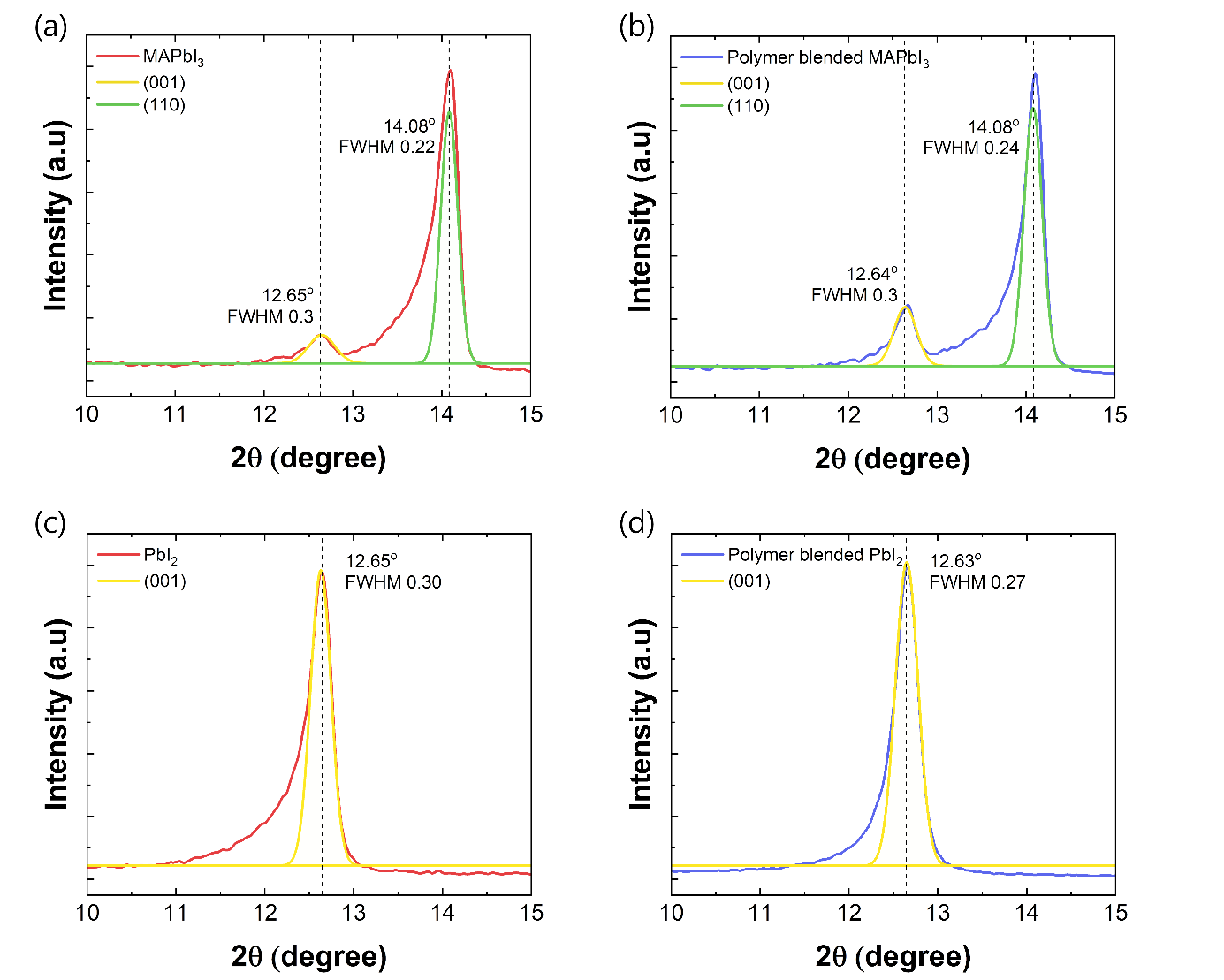
**

**Figure S3.** GIXRD measurements at an incident angle of 1.5° for (a) bare MAPbI_3_ and (b) polymer-blended MAPbI­_3_. Similar measurements were performed for (c) bare PbI_2_ and (d) polymer-blended PbI_2_. The analysis of the crystal structures of PbI_2_ and MAPbI_3_ films with polymer blending revealed no significant distortion in the crystal structure caused by the blended polymer.

**Section S4. *I-V* curve of bare MAPbI_3_**

**
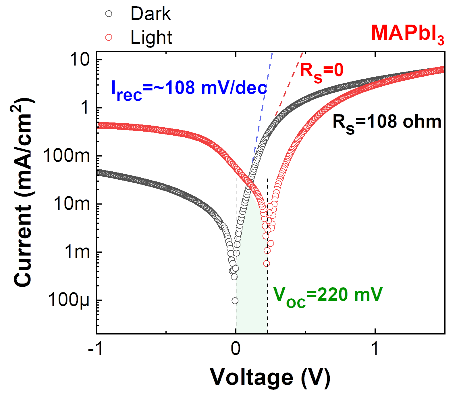
**

**Figure S4**. *I-V* curve of the bare MAPbI_3_ sample. The open-circuit voltage was measured at 220 mV, the recombination current at 108 mV·dec^–1^, and the parasitic resistance was found to be 108 ohms. These results indicate that the electrical properties of the bare MAPbI_3_ are relatively more stable compared to the polymer-blended perovskite sample.

**Section S5. Specifications of the Commercial SLD-70BG2 Si Photodiode**

| **Symbol** | **Parameter** | **Min** | **Typ** | **Max** | **Units** | **Test Conditions** |
| --- | --- | --- | --- | --- | --- | --- |
| *V*_OC_ | Open circuit voltage |  | 0.40 |  | V | *E_e_* = 25 mW·cm^–^­^2^ |
| *t*_R_ | Rise time |  | 4 |  | µs | *V_R_* = 5 V, *R_L_* = 1 kΩ |
| *t*_F_ | Fall time |  | 6 |  | µs | *V_R_* = 5 V, *R_L_* = 1 kΩ |
| *λ*_R_ | Sensitivity spectral range | 400 |  | 700 | nm |  |

Specifications of the commercial SLD-70BG2 Si photodiode

Section S5 presents the performance of the commercially available silicon photodiode used as the control group in this study. The commercial silicon photodiode used for comparison responds to light in the wavelength range of 400 nm to 700 nm, with a specific response to light at a wavelength of 450 nm employed in the experiments. The open-circuit voltage, measured through *I-V* characteristics under illumination with 25 mW·cm­^–2^ light, was found to be 0.4 V. Open-circuit voltages consistently measured in the range of 0.4 V to 0.5 V were verified. While the rising and falling times, as indicated in the datasheet, are specified in microseconds, these measurements were obtained under a 5 V reverse bias condition. In our TRNG system, the random number extraction process was conducted without an applied bias (0 V). Under conditions without reverse bias, the commercial Si photodiode maintained the turn-on state for several milliseconds even in the light-off state.

**Section S6. Output voltage distribution**

**
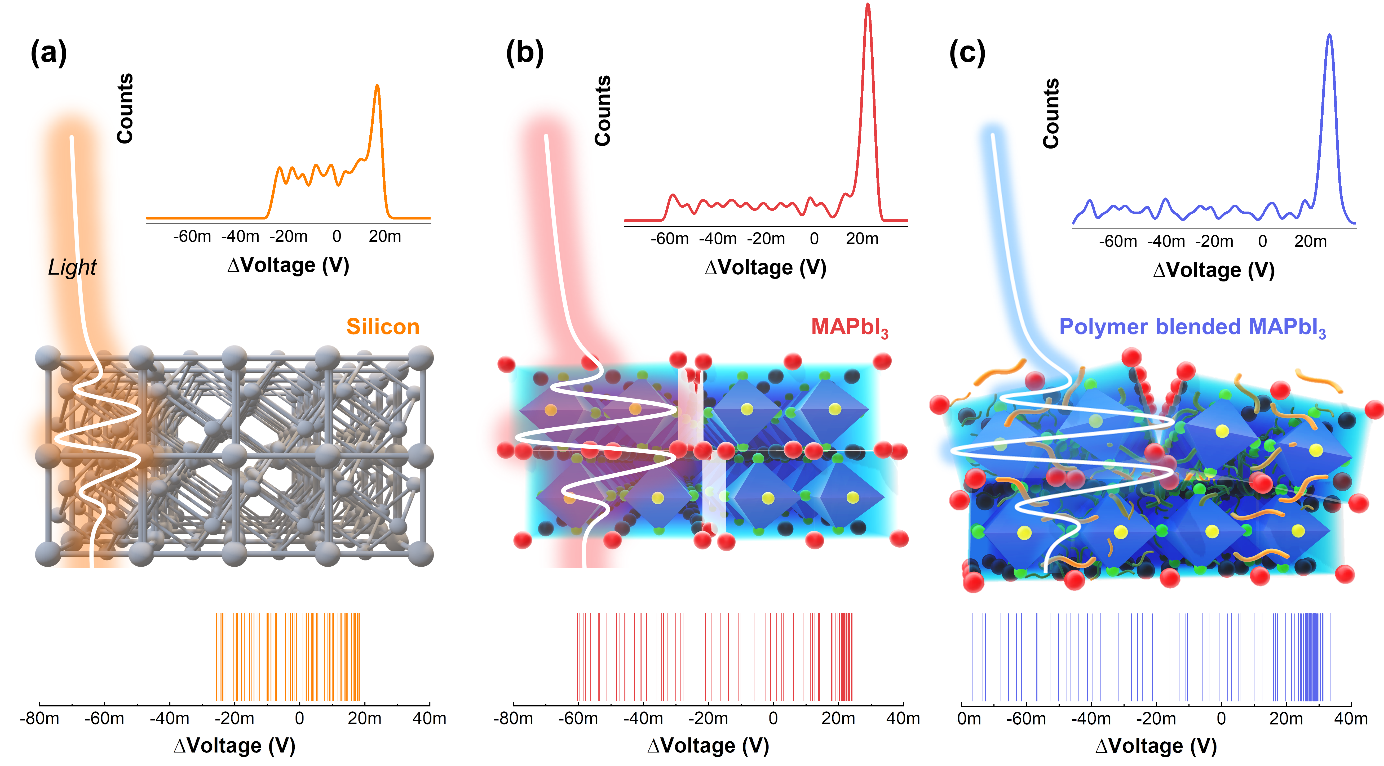
**

**Figure S5**. (a) Si, (b) MAPbI_3_, and (c) polymer-blended MAPbI_3_ photodetectors output voltage distribution induced by the same optical signal.

Figure S6 a-c. The differential voltage, denoted as ∆voltage, represents the deviation between the recorded output voltage and its mean value, indicating variability in both the amplitude and spectrum of output fluctuations. The graph overlaid on the crystal structure displays the distribution of output voltage errors in response to a consistent light signal input. Compared to the Si photodetector, both perovskite devices demonstrated a ∆voltage spanning a broader range, with a notably higher output density in the positive voltage domain. The minimum and maximum ∆voltage deviations were measured as 44 mV for the Si photodetector, 85.4 mV for the bare perovskite device, and 110.1 mV for the polymer-blended perovskite device, respectively.

**Section S7. Output bits generated by the TRNGs**

**
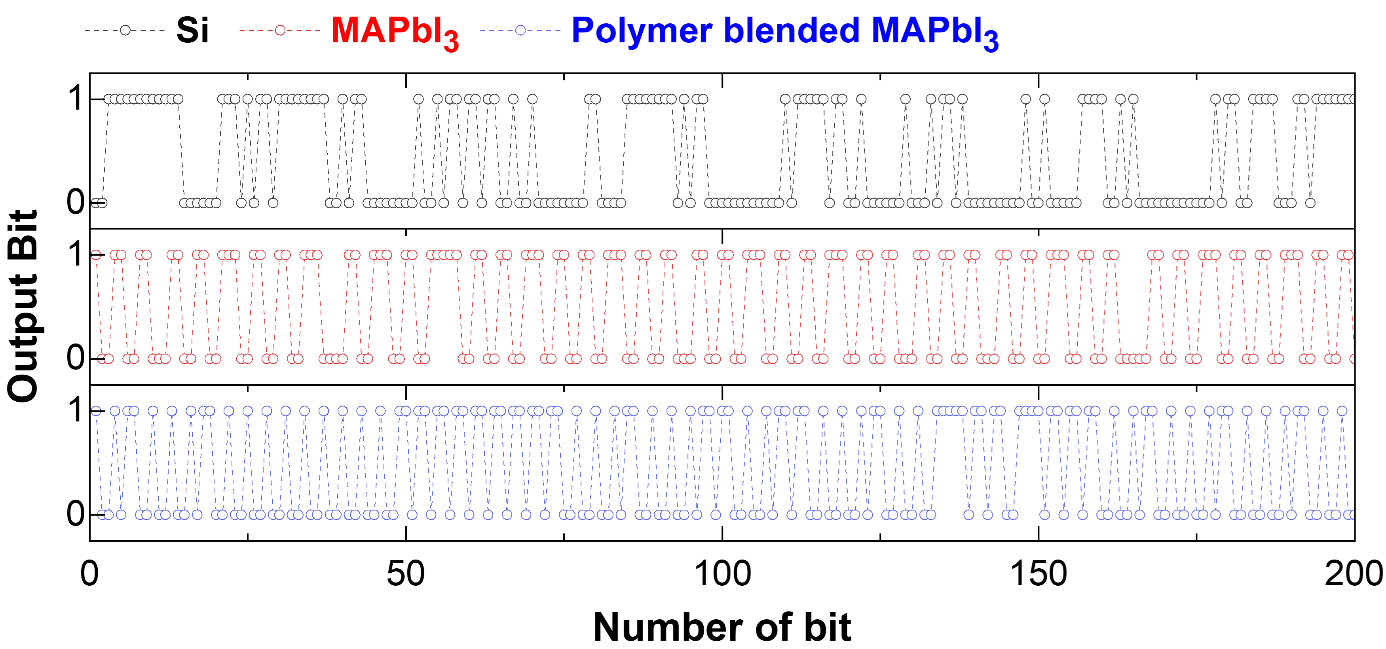
**

**Figure S6**. Output bits generated by the TRNGs (using the commercial Si photodiode, the bare MAPbI_3_, and the polymer-blended MAPbI_3_ photodetectors. The figure shows a fraction of the binary outcomes (200 out of 10^6^) after the JK flip-flop toggling. Binary numbers were assigned as “1” for voltages above and “0” for voltages below the mean voltage level detected at the JK flip-flop.

**Section S8. A circuitry of our TRNG system**

**
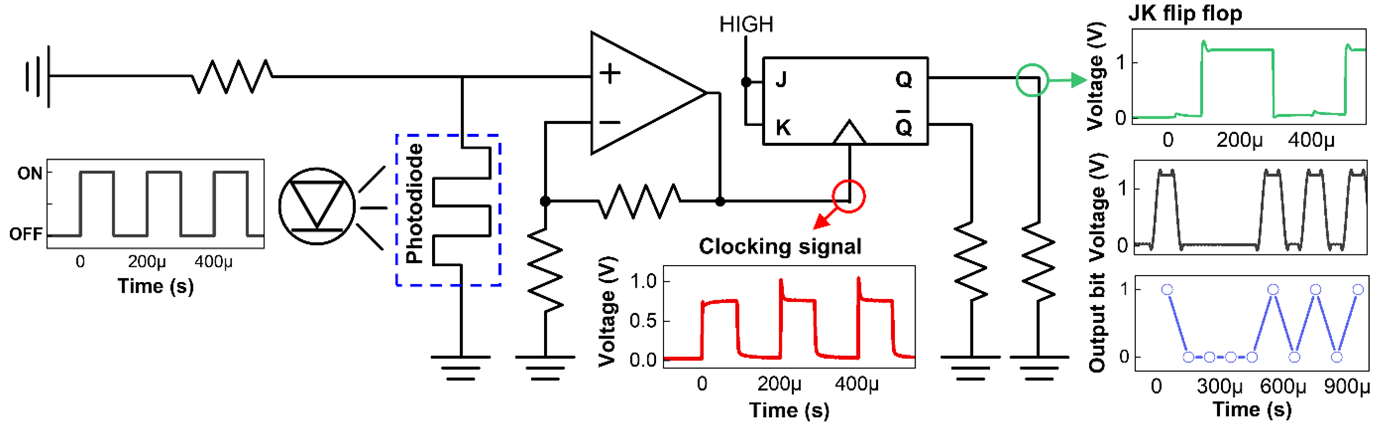
**

**Figure S7**. TRNG system schematic and extraction of random numbers through a JK flip-flop from a light-induced clocking signal.

**Section S9. Frequency dependence test for irradiation conditions**

**
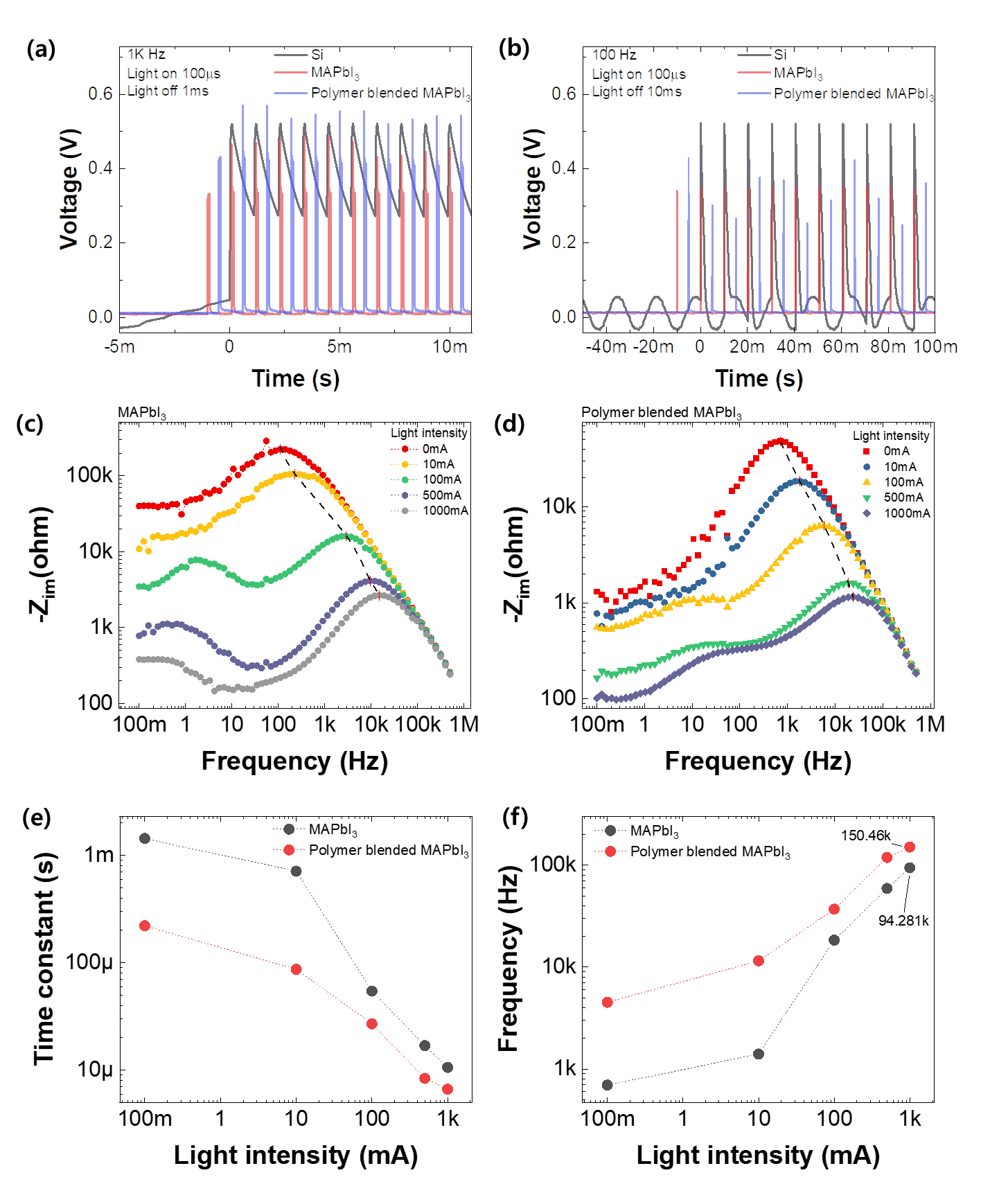
**

**Figure S8.** Frequency response characteristics of each photodiode material (Si, non-polymer-blended MAPbI_3_ and Polymer-blended MAPbI_3_) in the TRNG system at (a) 100 Hz and (b) 1 kHz. For the Si diode, the turn-on state was maintained at a light irradiation frequency of 1 kHz, which did not satisfy the condition for toggling the JK flip-flop. Therefore, the fastest operable frequency for the Si photodiode at 0 V bias is 100 Hz. (c) and (d) show the electrochemical impedance spectroscopy (EIS) measurement results of MAPbI_3_ and polymer-blended MAPbI_3_, respectively. In the EIS results, the frequency of the highest peak observed in the high-frequency range is calculated using the formula (τ = 1/2π f), as shown in Figure S10e. Under conditions of strong light irradiation at 1000 mA, the time constants of MAPbI_3_ and polymer-blended MAPbI_3_ were measured to be approximately 10 μs and 6.6 μs, respectively. Assuming this time constant represents one cycle, the frequency was calculated by taking the reciprocal, confirming the indirect operational times shown in (f).

**Section S10. Rising and falling time of the devices**


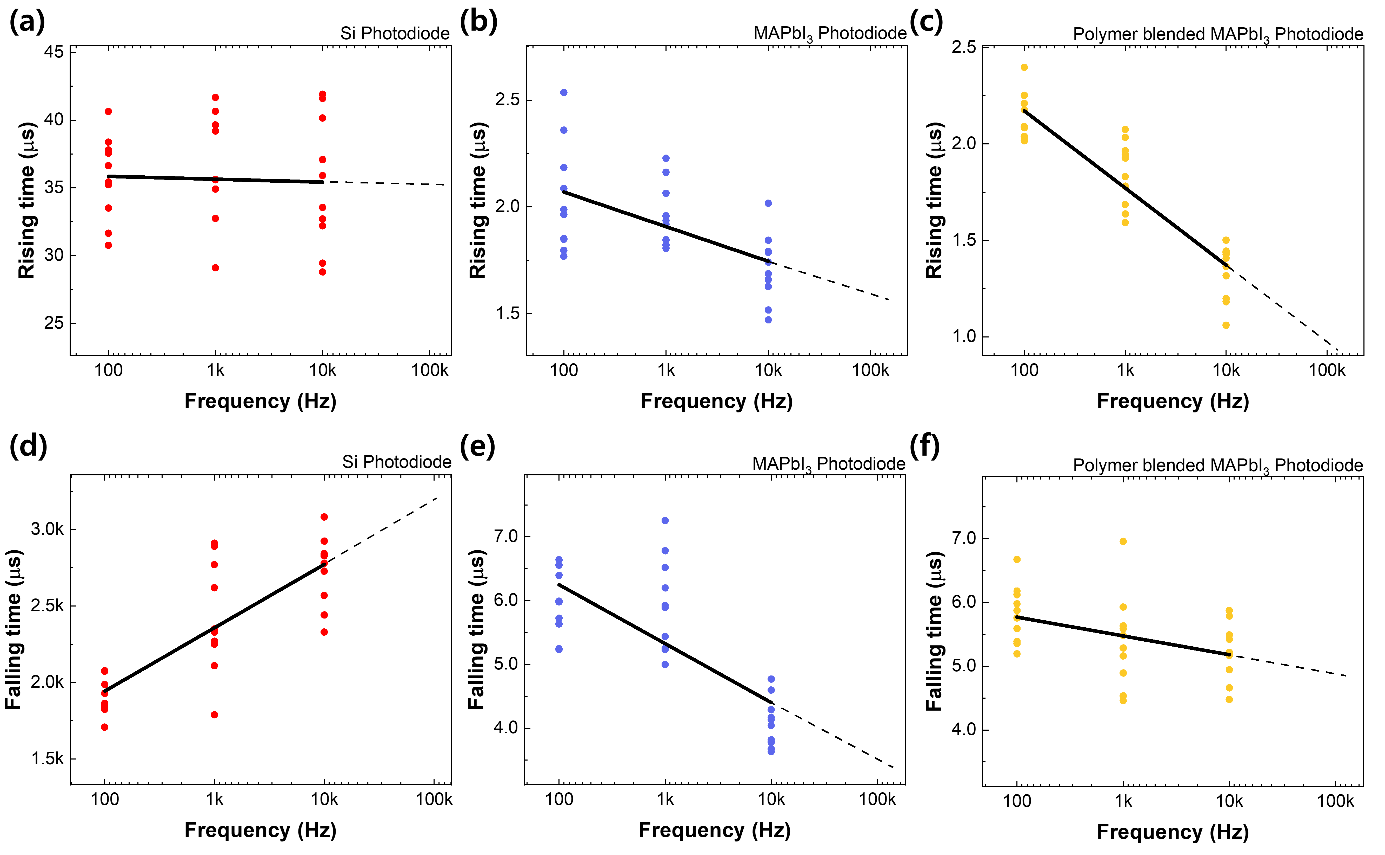


**Figure S9.** (a, d) Si photodiode, (b, e) MAPbI_3_ photodiode, and (c, f) polymer-blended MAPbI_3_ photodiode exhibiting (a-c) rising times and (d-f) falling times with respect to signal frequency, along with regression lines.

**Section S11. Clocking speed depending on the devices**

**
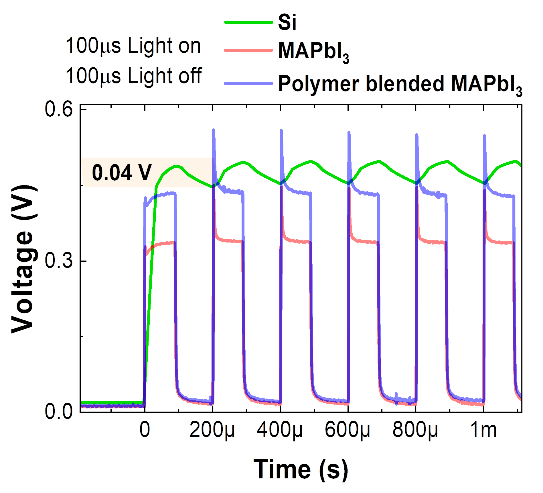
**

**Figure S10**. Measured clock signals in TRNG circuits with Si photodiode, bare MAPbI_3_, and polymer-blended MAPbI_3_ devices. Subjected to a 100 µs light pulse, the Si photodiode demonstrated a voltage amplitude of 0.04 V, failing to revert to the off state, which induced intermittent toggle events of the JK flip-flop with protracted delays.

**Section S12. NIST randomness test**

The P-values obtained by performing the 15 NIST tests are calculated by equations below.

**1. Frequency test within a block**

$P-value=igamc\left( \frac{N}{2},\frac{{4M\sum_{i=1}^{N} (\frac{\sum_{j=1}^{M} \varepsilon_{\left( i-1 \right)M+j}}{M}-\frac{1}{2})}^{2}}{2} \right)$ (eq. S1)

This test counts the percentage of M-bit blocks. It checks that the frequency of M-bit blocks is approximately M/2.

**2. Cumulative sums (forward/backwords)**

$P-value=1-\sum_{k=\left( \frac{-n}{z}+1 \right)/4}^{\left( \frac{n}{z}-1 \right)/4} \left[ \Phi\left( \frac{\left( 4k+1 \right)z}{\sqrt{n}} \right)-\Phi\left( \frac{\left( 4k-1 \right)z}{\sqrt{n}} \right) \right]+\sum_{k=\left( \frac{-n}{z}-3 \right)/4}^{\left( \frac{n}{z}-1 \right)/4} \left[ \Phi\left( \frac{\left( 4k+3 \right)z}{\sqrt{n}} \right)-\Phi\left( \frac{\left( 4k+1 \right)z}{\sqrt{n}} \right) \right]$ (eq. S2)

This test determines whether the cumulative sum of the partial sequences occurring in a sequence is too large or too small compared to the expected behavior of the cumulative sum for a random sequence.

**3. Runs test**

$P-value=erfc\left( \frac{\left| V_{n}(obs)-2n\pi(1-\frac{\sum_{j} \varepsilon_{j}}{n}) \right|}{2\sqrt{2n}\pi(1-\frac{\sum_{j} \varepsilon_{j}}{n})} \right)$ (eq. S3)

This test determines whether the oscillation between 0 and 1 in the number of total runs is too fast or too slow.

**4. Test for the longest run of ones in a block**

$P-value=igamc\left( \frac{K}{2},\frac{\sum_{i=0}^{K} \frac{\left( v_{i}-N\pi_{i} \right)^{2}}{N\pi_{i}}}{2} \right)$(eq. S4)

This test checks how well the length of the longest run observed in a block of M bits matches the length of the longest run expected in a random sequence

**5. Binary matrix rank test**

$P-value=e^{-\frac{\frac{{(F_{A}-0.2888N)}^{2}}{0.2888N}+\frac{{(F_{A-1}-0.5776N)}^{2}}{0.5776N}+\frac{{({N-F}_{A}{-F}_{A-1}-0.1336N)}^{2}}{0.1336N}}{2}}$ (eq. S5)

This test checks the rank of the heterogeneous submatrix in the entire sequence. Checks for linear dependence between strings.

**6. Discrete Fourier transform (spectral) test**

$P-value=erfc\left( \frac{\left| \frac{(N_{1}-N_{0})}{\sqrt{10\left( .95 \right)(.05)/4}} \right|}{\sqrt{2}} \right)$ (eq. S6)

This test detects periodic features in the sequence that indicate deviations. It detects how different the number of peaks above the 95% threshold is from 5%.

**7. Non-overlapping template matching test**

$P-value=\left( \frac{N}{2},\frac{\sum_{j=1}^{N} \frac{{(W_{j}-\mu)}^{2}}{\sigma^{2}}}{2} \right)$ (eq. S7)

This test checks for the number of occurrences of a pre-specified target string. It detects generators with too many occurrences of a given aperiodic pattern.

**8. Overlapping template matching test**

$P-value=igamc\left( \frac{5}{2},\frac{\sum_{i=0}^{5} \frac{{(v_{i}-N\pi_{i})}^{2}}{N\pi_{i}}}{2} \right)$ (eq. S8)

This test searches for a specific m-bit pattern by checking the number of occurrences of a pre-specified target string.

**9. Maurer’s “universal statistical” test**

$P-value=erfc\left( \left| \frac{\left( \frac{1}{K}\sum_{i=Q+1}^{Q+K} {log}_{2}(i-T_{j}) \right)-expectedValue(M)}{\sqrt{2}\left( 0.7-\frac{0.8}{M}+\left( 4+\frac{32}{M} \right)\frac{K^{-\frac{3}{M}}}{15} \right)\sqrt{\frac{var iance(M)}{K}}} \right| \right)$ (eq. S9)

This test checks the number of bits between matching patterns. It detects whether a sequence can be compressed sufficiently without losing information.

**10. Approximate entropy test**

$P-value=igamc\left( 2^{M-1},\frac{2n\left[ log2-\left( \varphi^{M}-\varphi^{M+1} \right) \right]}{2} \right)$ (eq. S10)

This test compares the frequency of overlapping blocks of two consecutive lengths to the expected result for a random sequence.

**11. Random excursions test**

$P-value=\left( \frac{5}{2},\frac{\sum_{k=0}^{5} \frac{\left( v_{k}\left( x \right)-J\pi_{k}\left( x \right) \right)^{2}}{J\pi_{k}\left( x \right)}}{2} \right)$ (eq. S11)

This test checks whether the number of visits to a particular state within a cycle deviates from the expected number for a random sequence.

**12. Random excursions variant test**

$P-value=erfc\left( \frac{\left| \xi\left( x \right)-J \right|}{\sqrt{2J(4\left| x \right|-2)}} \right)$ (eq. S12)

This test detects deviations from the expected number of visits to various states in a random walk.

**13. Serial test**

$P-value 1=igamc\left( 2^{m-2},\nabla\left( \left( \frac{2^{m}}{n}\sum_{i_{1}\ldots i_{m}} v_{i_{1}\ldots i_{m}}^{2}-n \right)-\left( \frac{2^{m-1}}{n}\sum_{i_{1}\ldots i_{m-1}} v_{i_{1}\ldots i_{m-1}}^{2}-n \right) \right) \right)$ (eq. S13)

$P-value 2=igamc\left( 2^{m-3}, \nabla^{2}\left( \left( \frac{2^{m}}{n}\sum_{i_{1\ldots}i_{m}} v_{i_{1\ldots}i_{m}}^{2}-n \right)-2\left( \frac{2^{m-1}}{n}\sum_{i_{1\ldots}i_{m-1}} v_{i_{1\ldots}i_{m-1}}^{2}-n \right)+\left( \frac{2^{m-2}}{n}\sum_{i_{1\ldots}i_{m-2}} v_{i_{1\ldots}i_{m-2}}^{2}-n \right) \right) \right)$ (eq. S14)

The test counts the frequency of all possible overlapping m-bit patterns across the entire sequence. It checks that this is approximately the same as expected for a random sequence.

**14. Linear complexity test**

$P-value=igamc\left( \frac{K}{2},\frac{\sum_{i=0}^{K} \frac{{(v_{i}-N\pi_{i})}^{2}}{N\pi_{i}}}{2} \right)$ (eq. S15)

This test checks the length of the linear feedback shift register (LFSR). It determines whether the sequence is complex enough to be considered random.

In the above equation, n is the length of the bit string. ε is the sequence of bits as generated by the TRNG being tested. m is the length in bits of the block. *N* is the number of blocks. *M* is the length of each block. *A* is the number of rows in each matrix. *V_n_* (obs) is the total number of runs across all n bits. *Φ* is the standard normal cumulative probability distribution function. *Q i*s the number of blocks in the initialization sequence. *N_0_* is 0.95 n/2. *N_1_* is the actual observed number of peaks in *M*. *ξ(x)* is the total number of times that state *x* occurred across all *J* cycles. *J* is the total number of zero crossings in *S’*. *K* and *π_i_* are determined according to the table in the manual. More information can be found in NIST’s a statistical test suite for random and pseudorandom number generators for cryptographic applications.

**Section S13. 10^6^ bit random numbers generated by the Si TRNG**


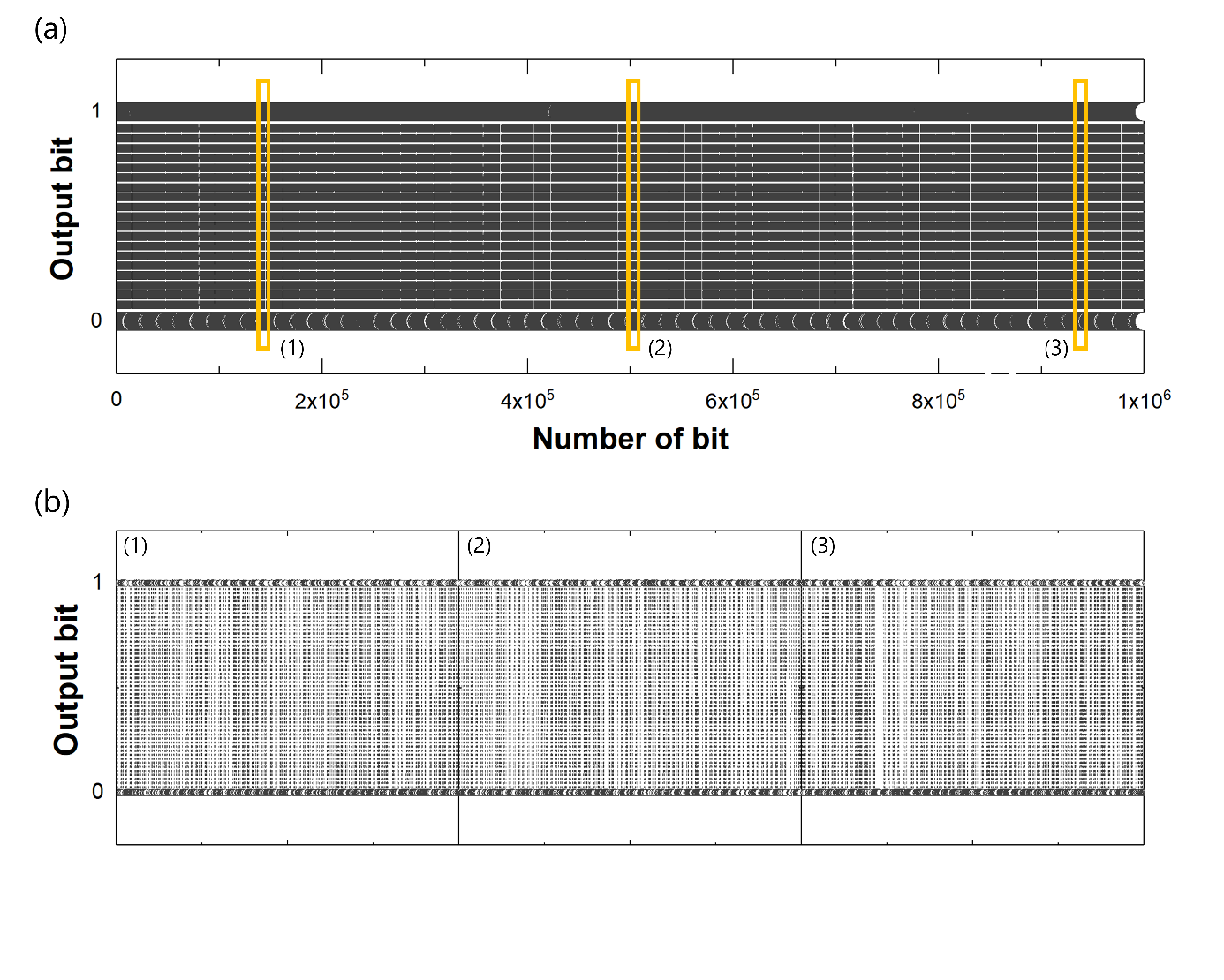


**Figure S11.** (a) 10^6^ bit operation test of the Si PD, (b) 10^3^ bit operation of the highlighted area in the upper figure’s yellow box.

**Section S14. 10^6^ bit random numbers generated by the bare MAPbI_3_ TRNG**


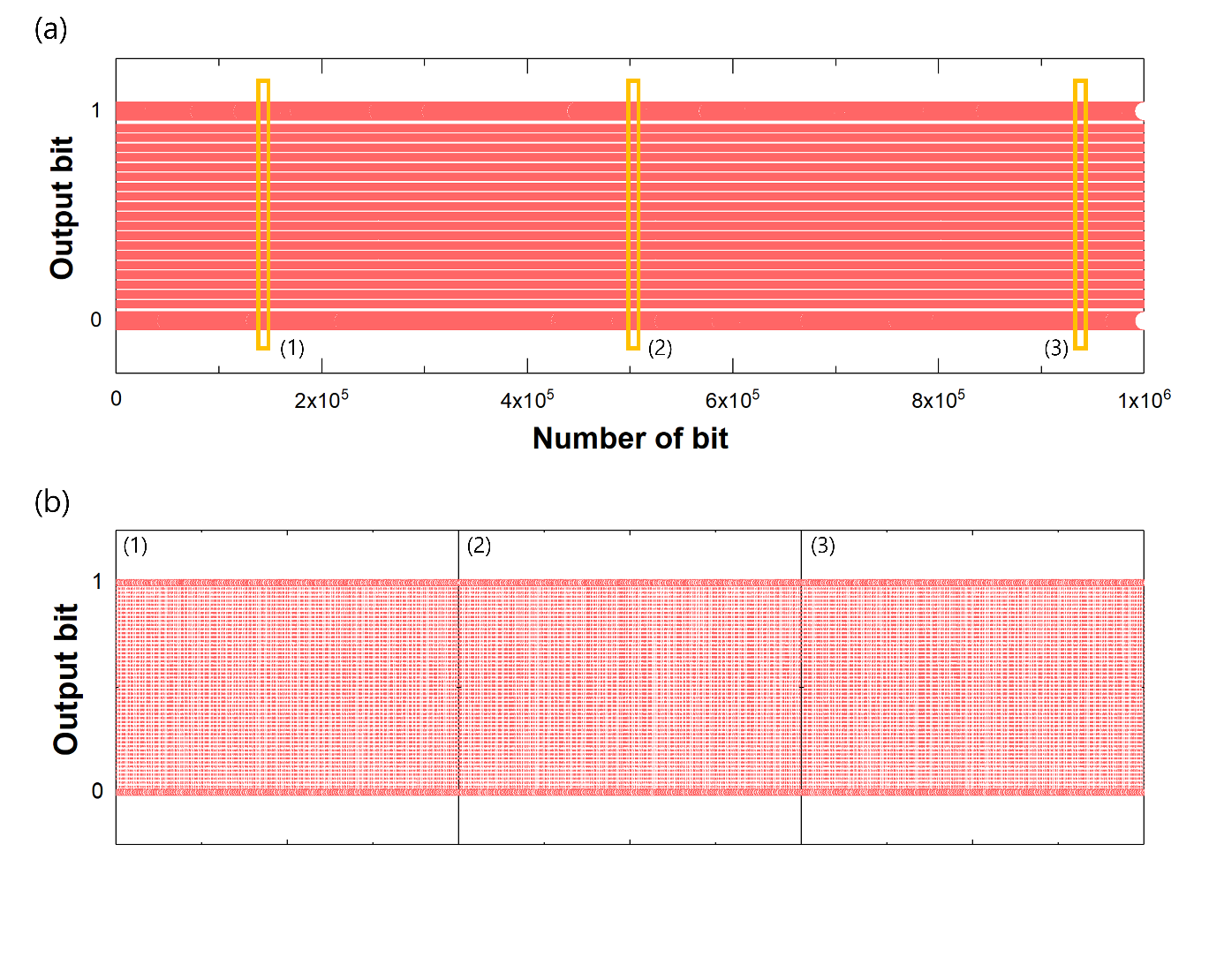


**Figure S12.** (a)10^6^ bit operation test of MAPbI_3_, (b) 10^3^ bit operation of the highlighted area in the upper figure’s yellow box.

**Section S15. 10^6^ bit random numbers generated by the polymer blended MAPbI_3_ TRNG**

**
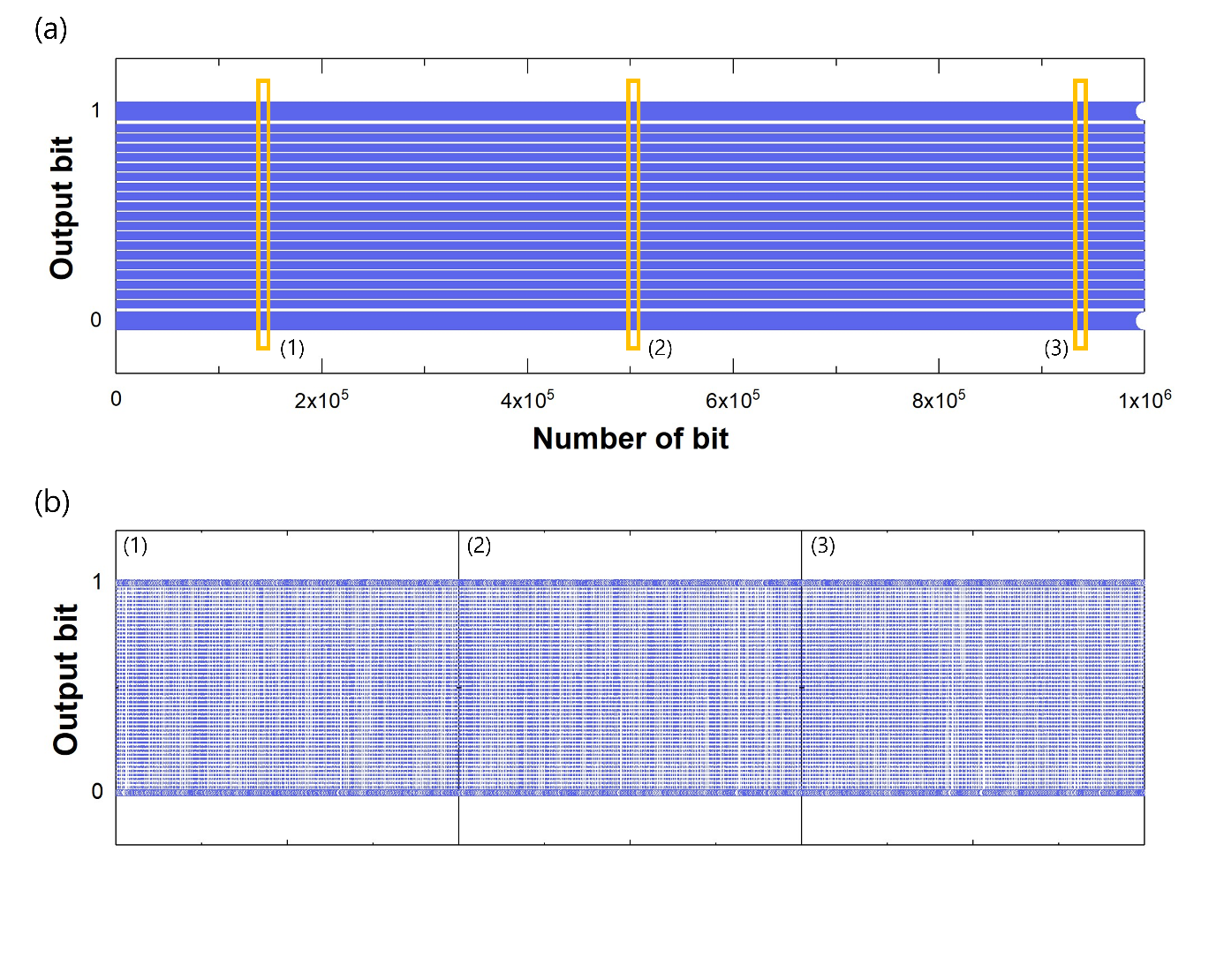
**

**Figure S13.** (a)10^6^ bit operation test of polymer-blended MAPbI_3_, (b) 10^3^ bit operation of the highlighted area in the upper figure’s yellow box.

**Section S16.** **480,000 random numbers extracted from TRNGs**


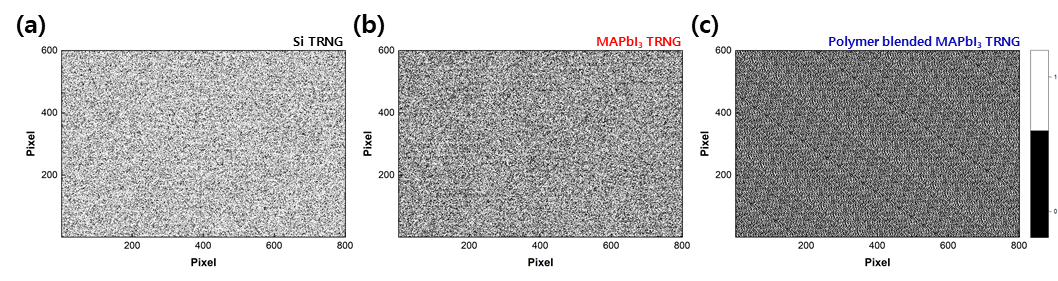


**Figure S14**. 480 000 random numbers extracted using (a) Si, (b) MAPbI_3_, and (c) polymer-blended MAPbI_3_ materials for image encryption.

**Section S17. Comparison of TRNG and QRNG Devices: Scalability, Integration, and Economic Feasibility**

| ref | Scalability | | | Ease of Integration | | Economic Feasibility |
| --- | --- | --- | --- | --- | --- | --- |
|  | Material | Fabrication | Flexibility | Input signal | Output signal | Cost per 1cm^2^ chip |
| This work | Polymer blended MAPbI_3_ | Solution process | 2%^[1]^ | Light | Electrical | $6.55 |
| ^[2]^ | NbO_x_ | Sputtering process | 1%^[3]^ | Thermal | Electrical | $211~$422 |
| ^[4]^ | HfO_2_ | Thermal Atomic Layer Deposition (ALD) | None | Electrical | Electrical | $215~$425 |
| ^[5]^ | ZrO_2_/BiFeO_3_ | RF Magnetron Sputtering | None | Electrical | Electrical | $90~$160 |
|  | Q-ROADM | Evaporation system | None | Light | Electrical | $12~$29 |
|  | QRNG | Evaporation system | None | Light | Electrical | $7~$15 |

**Table S1**. Comparison of scalability, integaration, and economic feasibility of TRNG and QRNG devices.

References

[1] K. H. Park, D. B. Kim, D. E. Lee, K. S. Park, Y. S. Cho, J. Alloys Compd. 2022, 908, 164607.

[2] G. Kim, J. H. In, Y. S. Kim, H. Rhee, W. Park, H. Song, J. Park, K. M. Kim, Nat. Commun. 2021, 12, 2906.

[3] J. M. Ang, P. A. Dananjaya, C. C. I. Ang, G. J. Lim, W. S. Lew, Sci. Rep. 2023, 13, 16000.

[4] K. S. Woo, Y. Wang, J. Kim, Y. Kim, Y. J. Kwon, J. H. Yoon, W. Kim, C. S. Hwang, Adv. Electron. Mater. 2019, 5, 1800543.

[5] Y. Jin, M. Zhu, Y. Zhou, Z. Zhang, J. Wang, X. Song, J. Meng, S. Ke, T. Zhang, S. Chen, R. Li, B. Jiang, C. Ye, Adv. Electron. Mater. 2024, 10, 2400072.
